# Effects of spin–orbit coupling on transmission and absorption of electromagnetic waves in strained armchair phosphorene nanoribbons

**DOI:** 10.1039/d3ra03686c

**Published:** 2023-07-24

**Authors:** H. Rezania, M. Abdi, E. Nourian, B. Astinchap

**Affiliations:** a Department of Physics, Razi University Kermanshah Iran rezania.hamed@gmail.com +98 831 427 4569 +98 831 427 4569; b Department of Physics, Faculty of Science, University of Kurdistan 66177-15175 Sanandaj Kurdistan Iran; c Research Center for Nanotechnology, University of Kurdistan 66177-15175 Sanandaj Kurdistan Iran

## Abstract

We compute the optical conductivity, both the imaginary and real parts of the dielectric constant, and the optical coefficients of armchair phosphorene nanoribbons under application of biaxial and uniaxial strains. The Kane–Mele model Hamiltonian has been applied to obtain the electronic band structure of phosphorene nanoribbons in the presence of a magnetic field. The effects of uniaxial and biaxial in-plane strain on the frequency behavior of the optical dielectric constant, and the frequency behavior of the optical absorption and refractive index of phosphorene nanoribbons have been studied, in terms of magnetic field, spin–orbit coupling and strain effects. Linear response theory and the Green’s function approach have been exploited to obtain the frequency behavior of the optical properties of the structure. Moreover, the transmissivity and reflectivity of electromagnetic waves between two media separated by a phosphorene-nanoribbon layer are determined. Our numerical results indicate that the frequency dependence of the optical absorption includes a peak due to applying a magnetic field. Moreover, the effects of both in-plane uniaxial and biaxial strains on the refractive index of single-layer phosphorene have been addressed. Also, the frequency dependence of the transmissivity and reflectivity of electromagnetic waves between two media separated by armchair phosphorene nanoribbons for normal incidence has been investigated in terms of the effects of magnetic field and strain parameters. Both compressive and tensile strain have been considered for the armchair phosphorene nanoribbons in order to study the optical properties of the structure. In particular, the control of the optical properties of phosphorene nanoribbons could lead to extensive applications of phosphorene in the optoelectronics industry. Also, such a study of the optical properties of phosphorene nanoribbons has further applications in light sensors. Meanwhile, the effects of spin–orbit coupling on the optical absorption and transmissivity of electromagnetic waves in phosphorene nanoribbons could be a novel topic in condensed-matter physics.

## Abstract 2

We compute the optical conductivity, both the imaginary and real parts of the dielectric constant, and the optical coefficients of armchair phosphorene nanoribbons under application of biaxial and uniaxial strains.

## Graphical abstract

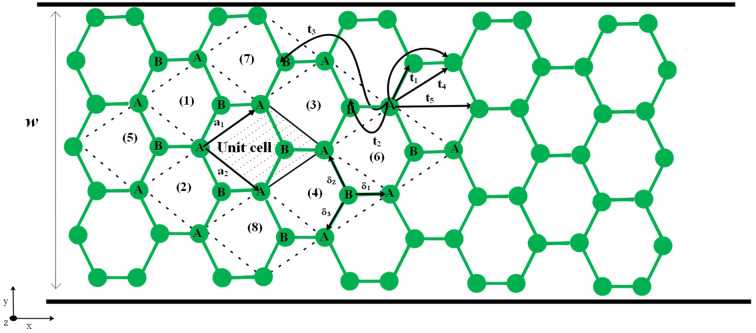

## 1 Introduction

Isolated quasi-two-dimensional black phosphorous, known as phosphorene, has attracted tremendous interest owing to its extraordinary electronic and optical properties in engineering applications.^1,2^ The sp^3^ hybridization leads to a puckered surface of the phosphorene layer, which generates a highly anisotropic band structure. A high carrier mobility of around 1000 cm^2^ V^−1^ s^−1^ has been predicted for phosphorene^3^ and a high on/off ratio of 10^4^ in phosphorene field-effect transistors at room temperature has been reported.^4^ Moreover, phosphorene layers may have unique potential thermoelectric applications.^5–7^ Studies on the optical and transport properties of monolayer phosphorene have demonstrated the existence of in-plane anisotropy in bulk phosphorene for two distinct zigzag and armchair directions. The nearly direct band gap of phosphorene increases with a decreasing number of layers, from 0.3 eV in the bulk to 2 eV for a monolayer.^8–11^ Such a large band gap of this two-dimensional nanomaterial results in significant applications in nanoelectronic, nanophotonic and optoelectronic devices.^12,13^ The phosphorene band gap can be controlled through various methods, like changing the temperature,^14^ strain,^15^ cutting the phosphorene into one-dimensional (1D) nanoribbons,^16,17^ and applying magnetic and electric fields.^18^ Strain is a powerful tool to control the electronic structure of phosphorene. This factor modifies the carrier mobility,^19^ tunes the band anisotropy,^20^ alters the transport properties,^21^ and induces variation in the optical conductivity.^22^ A critical strain can close the energy gap and turn phosphorene into a semi-Dirac semimetal material.^23–25^ The strain effects of the semi-Dirac semimetal material has already been observed experimentally.^26^ A high compressive strain can induce a structural phase transition or a gap transition with Dirac-like cones.^27^ The crystal structure of the phosphorene monolayer exhibits considerable flexibility for elastic planar strain.^27^ Also, the phosphorene plane has better elastic properties than graphene. For both zigzag and armchair directions, it can withstand a large strain of up to around 30 percent.^28,29^ The gapless band structure in the elastic limit^12,30,31^ of the phosphorene plane can arise from a considerable value of uniaxial strain in the direction normal to the phosphorene, so that a semiconductor–metal phase transition develops.^31–33^ Some theoretical studies on in-plane uniaxial strains effects on the band gap in phosphorene have demonstrated changing the electronic,^34^ thermoelectric^35^ and optical properties^36^ of this nanolattice structure.

The presence of edges in graphene has strong implications for the low-energy spectrum of the π-electrons.^37–39^ Some studies have been performed regarding the stabilization of the edge atoms of phosphorene by being terminated with hydrogen atoms.^40,41^ However, in our present work we have not considered the effects of hydrogen atoms located at the edges of the nanoribbon. We have assumed that the nanoribbon structure preserves its stabilization. The effects of hydrogen atoms as impurity atoms can be studied *via* an added Hamiltonian term in connection with the scattering of electrons from impurity hydrogen atoms.

Recent experiments using the mechanical method and the epitaxial growth^42^ method show it is possible to make phosphorene nanoribbons with various widths. Similar to graphene layers, the transport properties of phosphorene nanoribbons are affected by the edges of the structure.^43^ The electronic transport and optical properties of phosphorene nanoribbons with different edges, such as zigzag and armchair forms, have the extensive applications in future nanoelectronics.^44^ Selecting low-resistance metal contacts, where the Schottky-barrier height is small, increases electron injection efficiency.^45,46^ The edge dangling bonds of phosphorene nanoribbons can form intimate chemical bonding with the normal-metal electrode for charge transfer. Initial research on phosphorene nanoribbons was carried out by using numerical calculations, such as first-principles calculations, to mainly study the electronic properties of the phosphorene nanoribbons with the normal zigzag and armchair edges.^30,47,48^ The non-equilibrium Green’s function method has been used to study the transport properties of phosphorene nanoribbons and a dual-gate field-effect transistor has been proposed.^49^ It should be of great significance to investigate how to use phosphorene nanoribbons in the design of some electromagnetic devices.

The optical properties of single-layer phosphorene are of primary interest for the solar-cell industry, along with the tuning of these electro-optical features. This arises from intrinsic highly anisotropic electro-optical properties.^50–52^ Some results on the electronic properties of phosphorene layers show that the magneto-optical response can be tuned in the microwave-to-terahertz and visible frequency ranges, in contrast with a conventional two-dimensional electron gas.^53^ Based on such results, the optical conductivity of anisotropic phosphorene with spin–orbit coupling within the Kubo formalism shows that spin–orbit coupling changes the spin splitting.^53^ Also, the linear and optical absorption coefficients and relative refractive index changes as a function of the photon energy and magnetic field have been investigated, showing that the results are strongly influenced by the magnetic field.^54^ There are other materials that are utilized for microwave electromagnetic interference shielding.^55–60^ Electromagnetic-wave absorbing materials have thus attracted worldwide attention and are widely found in commercial and industrial settings, and improve electromagnetic interference shielding by effectively absorbing electromagnetic waves and converting them into other kinds of energy, such as thermal energy.^61,62^ The ideal electromagnetic absorbers should be relatively lightweight, highly thermally stable, capable of absorbing a wide range of electromagnetic frequencies, and cost effective.^63^ SiC and phosphorene, as samples of graphene-like structures, stand out for their unique properties in terms of electromagnetic wave absorption. SiC is a dielectric absorber by means of its intrinsic electric dipolar polarization. Also, it can be applied in harsh working environments with good electromagnetic absorption performance due to its thermal expansion, good thermal-shock resistance, high strength and good chemical inertness.^64,65^

Topological phase transitions in the phosphorene structure take place due to spin–orbit coupling and compressive biaxial in-plane strain.^66,67^ Such spin–orbit coupling arises from a perpendicular electric field or interaction with a substrate. Extensive theoretical studies have predicted the existence of a bulk gap in the band structure of the phosphorene plane and this band gap originates from both spin–orbit coupling and exchange-field factors.^68,69^ A simple model Hamiltonian to describe the physical properties of topological insulators has been proposed by Kane and Mele.^70^ This model Hamiltonian includes a tight binding term for the hopping amplitudes of electrons on the lattice sites of the structure and also an intrinsic spin–orbit coupling term for the honeycomb structures. Furthermore, a four-band tight-binding model with five neighbor hopping sites, considering the tuning effects of strains, is suitable for phosphorene nanoribbons.^71,72^ The Kane–Mele model essentially includes two copies, with different signs for the up and down spins, of a model introduced earlier by Haldane.^73^

The purpose of this paper is to apply a Kane–Mele model including intrinsic spin–orbit interaction for investigating the transmission and absorption of electromagnetic waves in armchair phosphorene nanoribbons. Also, simple tensile and compressive strains and a magnetic field have been applied to the phosphorene nanoribbon. The effects of uniaxial and biaxial in-plane strains on the optical properties of phosphorene are investigated using linear response theory in the context of the Kubo formula. Using a suitable hopping integral and in-plane strain parameters, the electronic band structure of electrons on the phosphorene nanoribbon has been calculated. We have obtained the density of states, absorption coefficient of electromagnetic waves, and optical coefficients. Also, we analyze the scattering of electromagnetic waves by a phosphorene nanoribbon located at the interface of two dielectrics and provide transmissivity and reflectivity curves. We study the effects of the magnetic field and the in-plane uniaxial- and biaxial-strain values along both zigzag and armchair directions on the frequency behavior of the absorption coefficient of electromagnetic waves, the optical coefficients and the dielectric function of armchair phosphorene nanoribbons. Also, we discuss and analyze how longitudinal magnetic field and strain values affect the transmissivity and reflectivity curves for a phosphorene nanoribbon located at the interface of two dielectric media.

Here, we add a few comments about the novelty of this present work. No research has been done on the optical conductivity, imaginary and real parts of the dielectric constant, and optical coefficients of armchair phosphorene nanoribbons using the Kane–Mele model. In this manuscript, we were able to report the optical properties of this structure with the Kane–Mele model. Also, by applying strain and a magnetic field, we controlled the optical properties of this structure, which provides the context for its application in the optoelectronics industry.

## 2 Model Hamiltonian and formalism

**Fig. 1:**
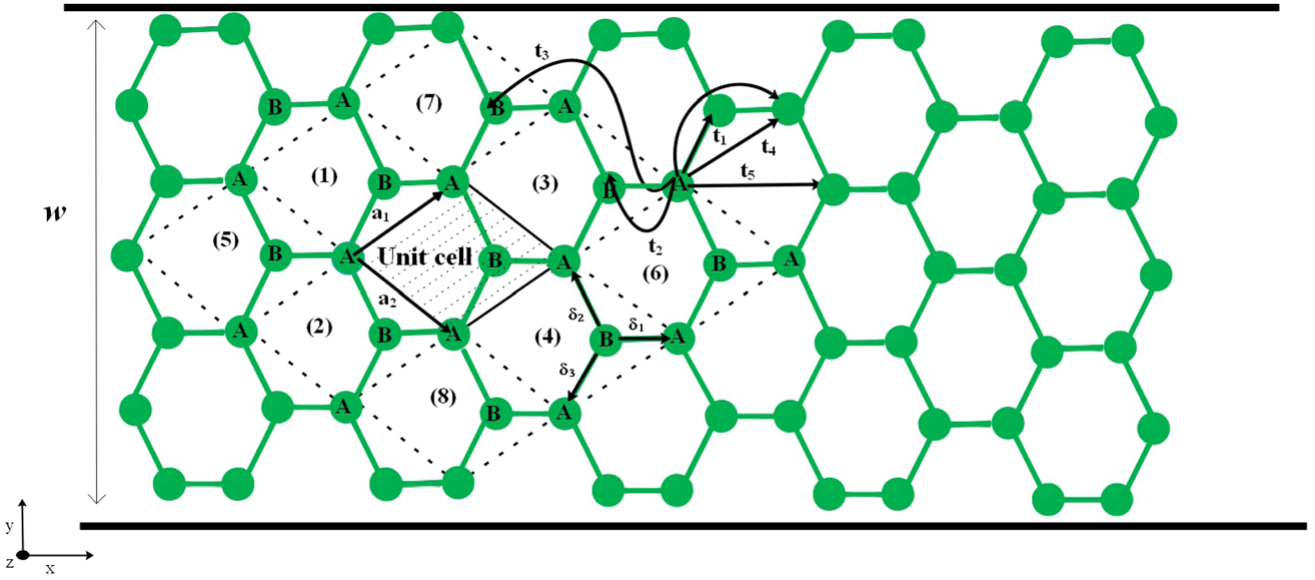
The crystal structure of an armchair phosphorene nanoribbon with the zigzag direction along the *x* axis. The various hopping amplitudes are shown. *w* is the ribbon width.

Here, we start with the geometric structure of an armchair phosphorene nanoribbon, including two types of sublattices, A and B, as shown in Fig. 1. The unit cell contains *w* A-type atoms and *w* B-type atoms. Based on the translational invariance, we choose the plane wave basis set along the *x* direction. The constructing vector of the unit cell in this lattice is shown by **a** = 3*a***i** where *a* is the bond length between phosphorous atoms. The Kane–Mele model Hamiltonian^70^ for armchair phosphorene nanoribbons, including the Zeeman term, is given by1
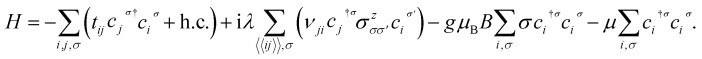


The first part in the model Hamiltonian is the tight binding model, so that *t*_*ij*_ describes the hopping integral energies between atomic lattice sites *i* and *j*. Only orbital p_*z*_ has been considered for the electrons in the tight-binding-model part of the model Hamiltonian. Previous studies on the hopping amplitudes in the phosphorene structure demonstrated that there are five different values for these hopping amplitudes.^71^ The numerical values for the hopping amplitudes of electrons between lattice sites in the phosphorene structure have been obtained as *t*_1_ = −1.220, *t*_2_ = 3.665, *t*_3_ = −0.205, *t*_4_ = −0.105 and *t*_5_ = −0.055 in units of eV.^71^ These hopping energy parameters have been indicated in Fig. 1. In eqn (1), *C*_*i*_^*σ*^ is the annihilation operator of an electron with spin *σ* in lattice site *i*. The second term in the model Hamiltonian in eqn (1) describes spin–orbit coupling, so that the coupling strength is denoted by the parameter *λ*. It should be noted that this term has finite values for next-neighbor lattice sites 〈〈*ij*〉〉. The third Pauli matrix is *σ*^*z*^. Depending on the orientation of the sites, the values of *ν*_*ji*_ are obtained as *ν*_*ji*_ = ±1. A standard statement for *ν*_*ji*_ is *ν*_*ji*_ = (**d**_*j*_ × **d**_*i*_)_*z*_ = ±1, where **d**_*j*_ and **d**_*i*_ are the two unit vectors along the nearest-neighbor bonds connecting site *i* to its next-nearest neighbor, *j*. The third term refers to the Zeeman term due to interaction between the spin degrees of freedom of the electrons and the external longitudinal magnetic field. In this part, *g* indicates the gyromagnetic constant and *μ*_B_ denotes the Bohr magneton constant. *B* denotes the magnetic field strength for the applied magnetic field perpendicular to the plane of the phosphorene nanoribbon.

In order to present the matrix form of the model Hamiltonian in eqn (1) for the nanoribbon structure, we introduce the Bloch–Hilbert space basis as |*α*, *k*_*x*_, *p*〉, which is expanded in terms of orbital wave function basis |*α*, *l*, *m*〉. This expansion has been proposed as^74^2
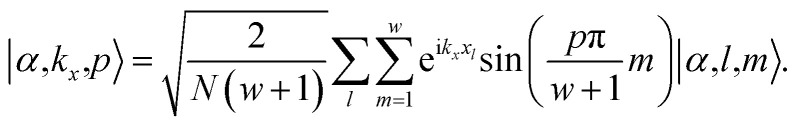
where *x*_*l*_ introduces the position of the *l*-th unit cell along the *x* direction, as shown in Fig. 1. *w* denotes the width of the ribbon and *N* is the number of unit cells along *x* direction. *α* indicates the sublattice index as *α* = *A*,*B* and the wave vector 
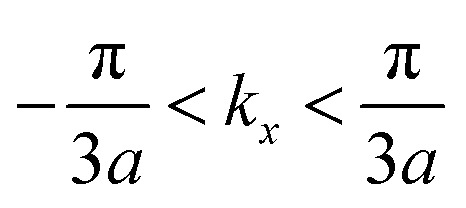
 belongs to the first Brillouin zone of an atomic chain with lattice constant 3*a* (see Fig. 1). The quantum number *p* is introduced in the following. The function 
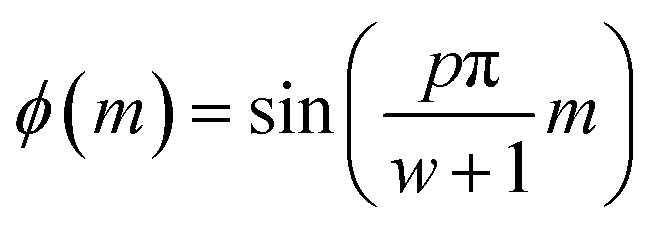
 should satisfy the hard-wall boundary condition, *i.e. ϕ*(*w* + 1) = *ϕ*(0) = 0.^74^ Moreover, the hard-wall boundary condition implies the quantum number *p* = 1, 2, …, *w*.

Using the Bloch–Hilbert space basis introduced in the matrix form of the model Hamiltonian in eqn (1) is written as follows:3
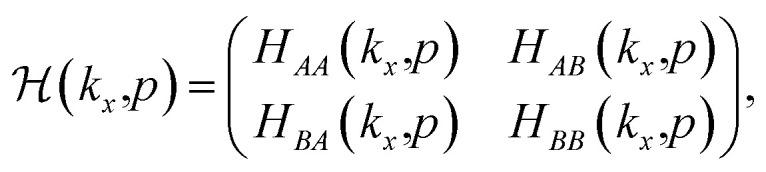
so that the matrix elements of *H*_*αβ*_(*k*_*x*_,*p*) with *α*,*β* = *A*,*B* are given by4
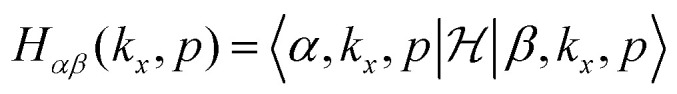


The matrix elements of *H*_*αβ*_ are expressed based on hopping amplitudes *t*_1_, *t*_2_, *t*_3_, *t*_4_ and *t*_5_ and spin–orbit coupling *λ*. The diagonal matrix elements 
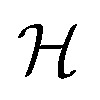
 in eqn (3) are written in terms of the hopping amplitude of electrons between next-nearest-neighbor lattice sites on the same sublattice and *λ*. Meanwhile, the off-diagonal matrix elements *H*_*AB*_ and *H*_*BA*_ are stated based on the hopping amplitude of electrons between nearest-neighbor atoms and next-nearest-neighbor atoms on different sublattices. These matrix elements are given by5
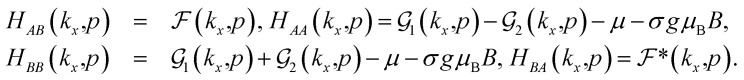
with6
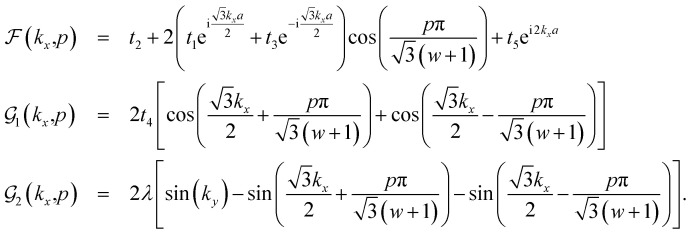


The first two nearest-neighbor hopping parameters have remarkable values compared to the others, and in general play the main role in constructing the electronic structure. The matrix elements of the model Hamiltonian in eqn (5) and (6) lead to the following electronic band structure of the electrons for the armchair phosphorene nanoribbon:7
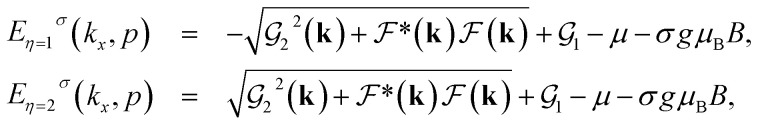
where *η* labels the band index for valence or conduction band index and *σ* = ↑, ↓ denotes the quantum number of the spin angular momentum of an electron. The chemical potential, *μ*, can be determined *via* the relation between the concentration of electrons (*n*_e_) and chemical potential:8
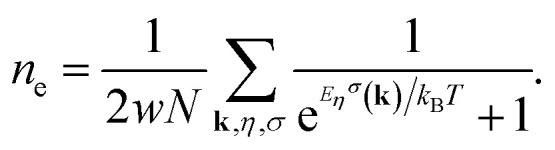
*k*_B_ is the Boltzmann constant. Based on the values of electronic concentration *n*_e_, the chemical potential values are obtained.

We can rewrite the model Hamiltonian in eqn (1) based on the Hilbert space of the band index *η* as9
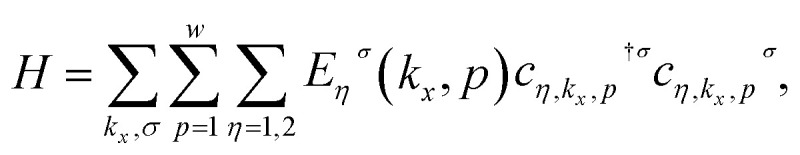
where 
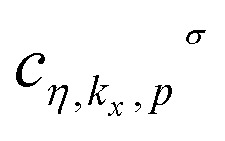
 defines the creation operator of an electron with spin *σ* in band index *η* at wave vector *k*_*x*_ belonging to the first Brillouin zone of the structure with quantum number *p*. Using the model Hamiltonian in eqn (9), the frequency Fourier transformation of Green’s function is given by^79^10
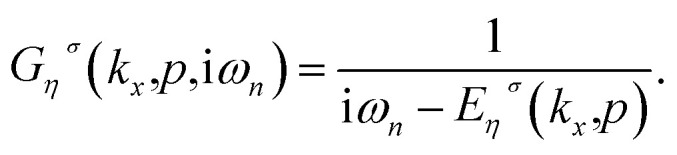
Here *ω*_*n*_ = (2*n* + 1)π*k*_B_*T* denotes the fermionic Matsubara frequency in which *T* is the equilibrium temperature. Using the electronic band structure spectrum in eqn (7), we can obtain the density of states of the armchair phosphorene nanoribbon due to spin–orbit interaction and an external magnetic field as11
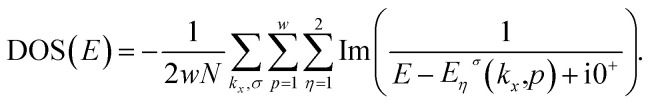


The density of states (DOS) includes prominent asymmetric peaks due to the band edge of parabolic subbands. The peak positions arise from the band-edge state energies and the density of states heights are proportional to the inverse square root of the subband curvature and band degeneracy.

Since the phosphorene nanoribbon can sustain a tensile strain of up to about 30 percent along the armchair or zigzag direction, this structure has much better elastic deformation properties than graphene sheets. Under the different types of strains, the bond length and thus the hopping energy are changed for phosphorene,^75,76^ so that applying strain leads to alterations of the hopping amplitudes, *t*_*ij*_. According to the Harrison rule, the hopping amplitudes for p orbitals are proportional to the inverse square of the atomic distance, *i.e.*, *t*_*i*_ ∝ |**r**_*i*_|^−2^ with *i* = 1, 2, 3, 4, 5. Under application of strain to the phosphorene nanoribbon and in the linear deformation regime, the deformed bond vector 
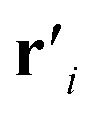
 can be expanded in terms of the undeformed bond vector **r**_*i*_ as follows:12
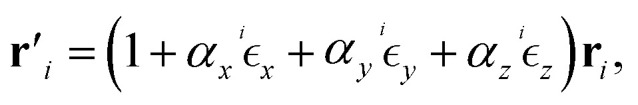
where we have the expressions 
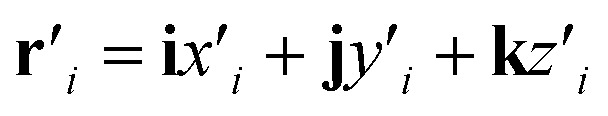
 and **r**_*i*_ = **i***x*_*i*_ + **j***y*_*i*_ + **k***z*_*i*_. Also, *ε*_*β*=*x*,*y*,*z*_ is the strain modulus along the *β* direction. For each hopping amplitude *t*_*i*_, with *i* = 1, 2, 3, 4, 5 in phosphorene structure, the values of the dimensionless geometrical coefficients have been obtained^71,77,78^ as 
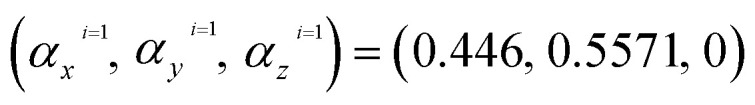
, 
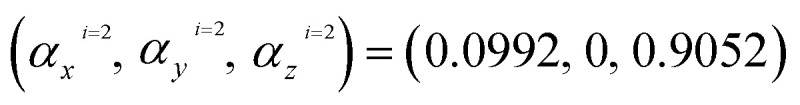
, 
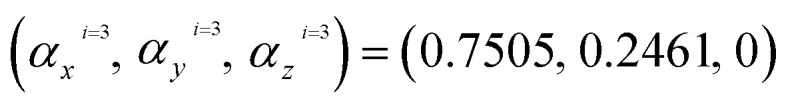
, 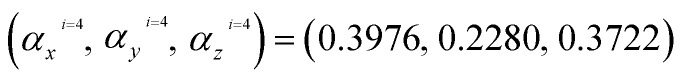 and 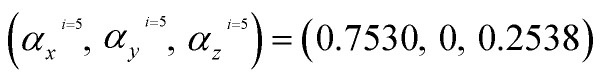. Based on eqn (12) and according to the Harrison rule, the hopping energies of the strained phosphorene nanoribbon, 
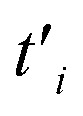
, are obtained using13
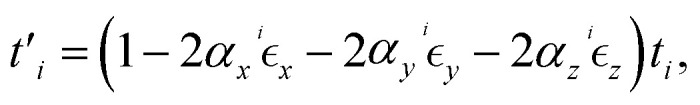
where *t*_*i*_ describes the hopping energies of the pristine phosphorene nanoribbon structure. Thus, the physical properties of strained phosphorene have been obtained by using the electronic band structure of the deformed phosphorene structure. This band structure of deformed phosphorene can be readily found by replacing 
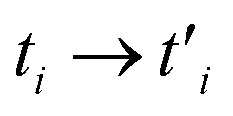
 in eqn (6).

## 3 Absorption coefficient and transmission of electromagnetic waves

In this section, the Kubo linear response theory^79^ has been used for calculating the real part of the in-plane optical conductivity of armchair phosphorene nanoribbons. Based on the optical conductivity, we have found both real and imaginary parts of the dielectric functions, the optical coefficients and the absorption coefficient of electromagnetic waves in the structure. An electromagnetic field with electric field polarization along the zigzag direction (see Fig. 1) has been applied to the armchair phosphorene nanoribbon. Such an applied electric field leads to the Hamiltonian term **J**_e_·**A**, which is added to the original Hamiltonian *H* in eqn (9). In this added term, **A** denotes the vector potential, which can be obtained from the external electric field, **E**, of the radiated electromagnetic field *via*
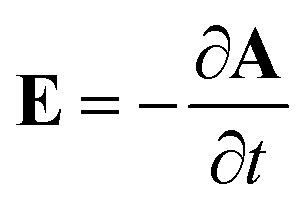
, and **J**_e_ refers to electrical current. The optical conductivity *σ*(*ω*) is obtained as the response of the electrical current density (**J**_e_) to an external electrical field. According to response theory, the current density, **J**_e_, along the spatial *α* direction is related to the *β* component of the external electric field, *E*_*β*_, using the expression **J**_e_^*α*^(*ω*) = *σ*_*αβ*_(*ω*)*E*_*β*_(*ω*).

Based on the continuity equation for the electrical charge current density, **J**_e_, the explicit form of the electrical current operator for phosphorene nanoribbons can be obtained from the bilinear form of the model Hamiltonian in eqn (9). The operator form of the electrical charge current density of operator **J**_e_ along the *x* direction for itinerant electrons of phosphorene nanoribbons, in the context of the Kane–Mele model Hamiltonian, is given by14
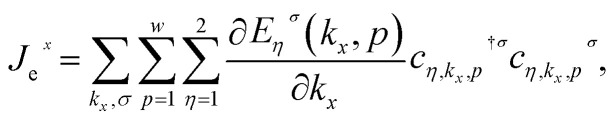


The linear response theory is implemented to obtain the optical conductivity under the assumption of a low dynamic electric field (as a perturbing field). The Kubo formula gives the optical conductivity, *σ*(*ω*), in terms of a correlation function of electrical current operators:15
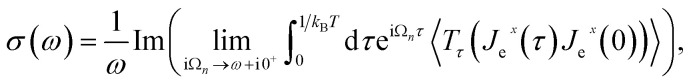
*τ* is the imaginary time and *T* denotes the time ordered product. Where it is assumed that the electrical current flows along the zigzag direction. Ω_*n*_ = 2*n*π*k*_B_*T* with integer *n* refers to the bosonic Matsubara frequency. After substituting eqn (14) into eqn (15) within an linear response approximation, the correlation functions between current operators can be obtained as16
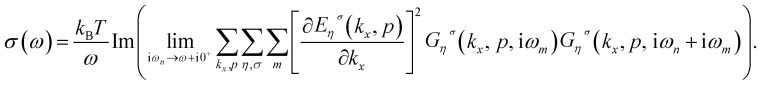
According to the Lehmann representation,^79^ the imaginary part of the retarded Green’s function and the Matsubara form of Green’s function are related to each other as17
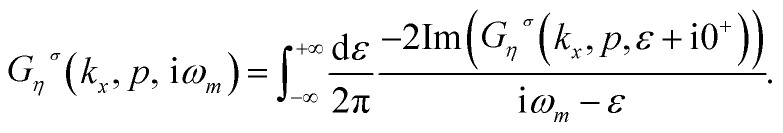


Using the Lehmann representation and after summation over Matsubara’s fermionic frequency, *ω*_*m*_, we can arrive at the following relation for the optical conductivity, *σ*(*ω*), of armchair phosphorene nanoribbons as18
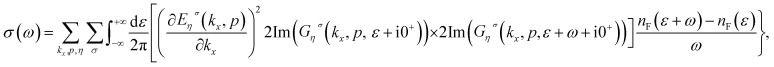
where 
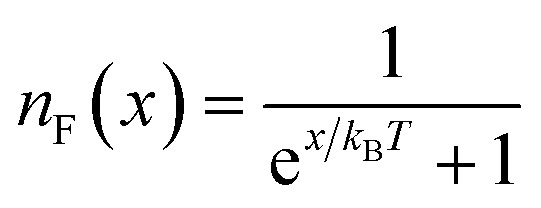
 is the Fermi–Dirac distribution function and *T* denotes the equilibrium temperature. Substituting the electronic Green’s function of armchair phosphorene nanoribbons presented in eqn (10) into eqn (18) and performing numerical integration over the wave vector through the first Brillouin zone, the results of optical absorption in terms of photon frequency, *ω*, have been obtained. Here, the contribution of both inter- and intraband transitions to the optical conductivity in eqn (18) has been considered. The dielectric function of the phosphorene nanoribbon is introduced by *ε*(*ω*) = *ε*_1_(*ω*) + i*ε*_2_(*ω*). The imaginary part of the dielectric function of the phosphorene nanoribbon, *i.e. ε*_2_(*ω*), is related to the dynamic optical conductivity *via*19
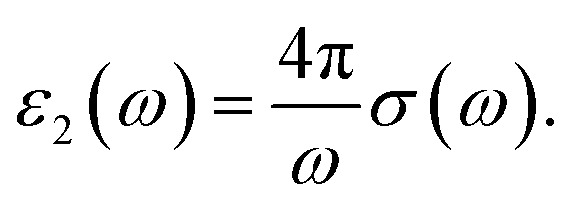


The real part of optical dielectric function *ε*_1_(*ω*) can be obtained from *ε*_2_(*ω*) using the Kramers–Kronig relation:^80^20
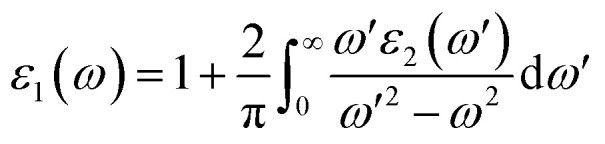


The complex optical coefficient of the phosphorene nanoribbon is defined as *N*(*ω*) = *n*(*ω*) + i*k*(*ω*) where *n* is the ordinary refractive index and *k* refers to the extinction coefficient. *n* and *k* can be rewritten in terms of *ε*_1_(*ω*) and *ε*_2_(*ω*) as21
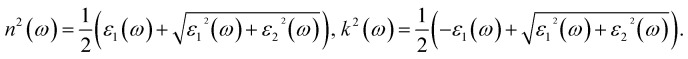


**Fig. 2:**
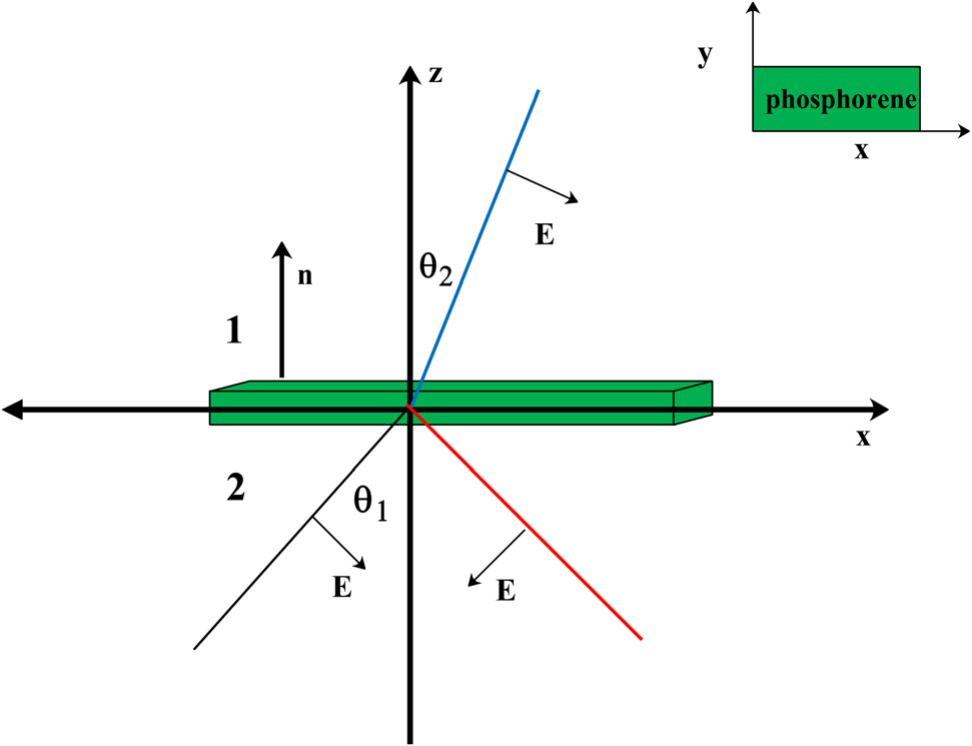
Geometry of polarized electromagnetic wave scattering between two media with phosphorene separating them. The electrical permittivities of the two media are *κ*_*i*_*ε*_0_ with *i* = 1, 2.

The absorption coefficient, *α*(*ω*), which is proportional to the rate of energy dissipation of an electromagnetic wave in the phosphorene nanoribbon, is obtained from *k*(*ω*) by the relation22
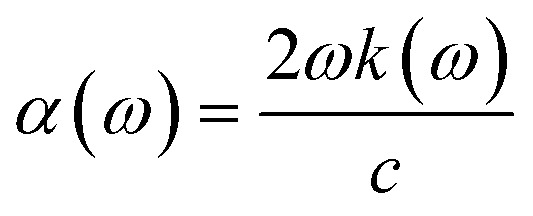
in which *c* is the velocity of light in the vacuum. In the following, we obtain the reflectivity and the transmissivity of electromagnetic waves between two media separated by a phosphorene nanoribbon. The electrical permittivity of each medium is given by *κ*_*i*_*ε*_0_ with *i* = 1, 2; *κ* is the electrical permittivity constant and *ε*_0_ is the perimittivty of the vaccum. We assume the normal vector of the monolayer is along the *z* direction. Furthermore, the propagation direction of the field is considered to be **k** = (*k*_*x*_, 0, *k*_*z*_) and the polarization of the field is given by **E** = (*E*_*x*_, 0, *E*_*z*_). The schematic representation of the scattering geometry has been plotted in Fig. 2. Using electromagnetic boundary conditions for the electric field and displacement field and the continuity equation in momentum space, we arrive at the following result for the transmissivity, *T_t_*, for normal incidence with propagation direction **k** = (0, 0, *k*_*z*_) and incident angle *θ*_1_ = 0 in Fig. (2) (ref. 81):23
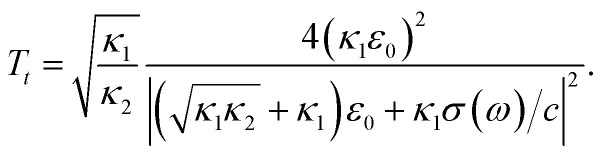


Also, for normal incidence the reflectivity is as follows:^81^24
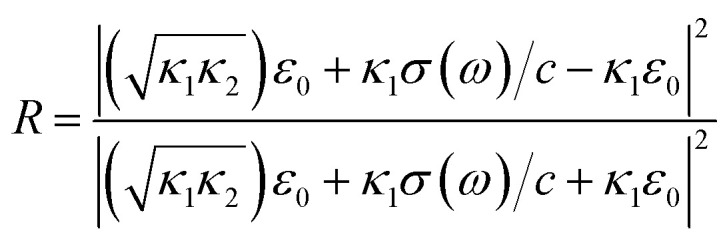
where *σ*(*ω*) is the dynamic optical conductivity of the armchair phosphorene nanoribbon, whose relation has been given in eqn (18). By substituting the optical conductivity presented in eqn (18) into eqn (23) and (24), the transmission and reflection coefficients have been obtained, respectively.

In the next section, we mention the numerical results for the absorption coefficient and refractive index of armchair phosphorene nanoribbons in the presence of strains and magnetic field effects. Also, the numerical results of the reflectivity and the transmissivity of electromagnetic waves between two media separated by a phosphorene nanoribbon have been also studied.

## 4 Numerical results and discussion

In this section, our numerical results for the absorption rate of electromagnetic waves in armchair phosphorene nanoribbons in the presence of a magnetic field and spin–orbit coupling effects are presented. Also, we have studied the photon frequency dependence of the refractive index and extinction coefficient. The effects of biaxial and uniaxial strains on the frequency dependence of the transmission and reflection coefficients of electromagnetic waves from phosphorene nanoribbons have been investigated. Both positive (tensile) and negative (compressive) strain parameters are considered in our results for the optical properties of phosphorene nanoribbons. We have focused on the frequency dependence of the real and imaginary parts of the dielectric function of phosphorene nanoribbons in the presence of biaxial and uniaxial strain effects. It should be noted that we set the chemical potential *μ* = 0 in all calculated quantities, so that we have considered the half-filling case for the phosphorene layer. Moreover, the width of the ribbon has been assumed to be *w* = 7 in our numerical results. The spin–orbit coupling strength is considered to be *λ* = 0.2 eV. According to dimensionless geometrical coefficients *αj*^*i*^ with *i* = 1, 2,…5 and *j* = *x*, *y*, *z*, the hopping amplitudes of the strained phosphorene nanoribbon, *i.e.*
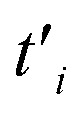
, have been given in eqn (13). With substitution of 
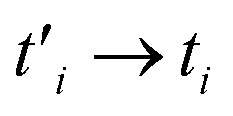
 into eqn (6), the matrix elements of the model Hamiltonian have been found. Afterwards, we can obtain the band structure of the strained armchair phosphorene nanoribbon in the presence of a magnetic field and spin–orbit coupling using eqn (7). The density of states and optical conductivity *σ*(*ω*) are found by substitution of Green’s function presented in eqn (10) into eqn (18). Finally, the frequency dependence of the optical properties of the strained armchair phosphorene nanoribbon are derived by relations in eqn (19)–(24). The electric field polarization of the electromagnetic wave is assumed to be along the zigzag direction so that the propagation direction of the electromagnetic wave is perpendicular to the plane of the phosphorene layer. Moreover, the temperature has been fixed at *T* = 300 K in all the following numerical results.

**Fig. 3:**
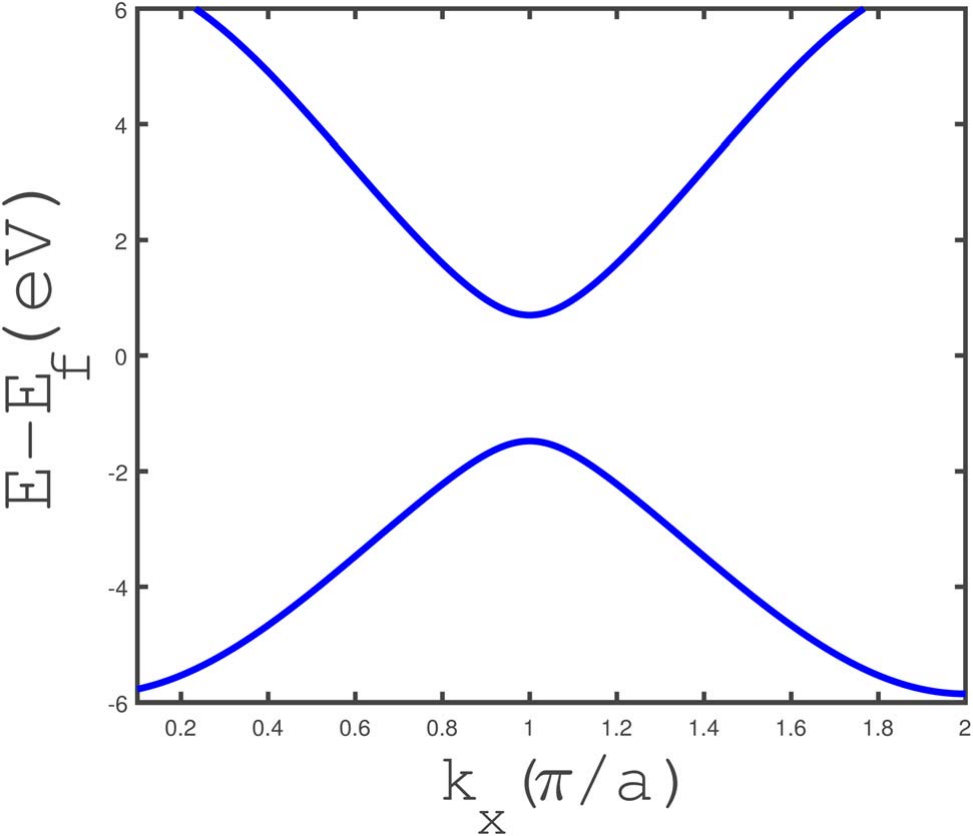
The band structure of the phosphorene armchair nanoribbon in terms of wave vector belonging to the first Brillouin zone, in the absence of a magnetic field and strain parameter. The ribbon width is considered to be *w* = 7.

**Fig. 4:**
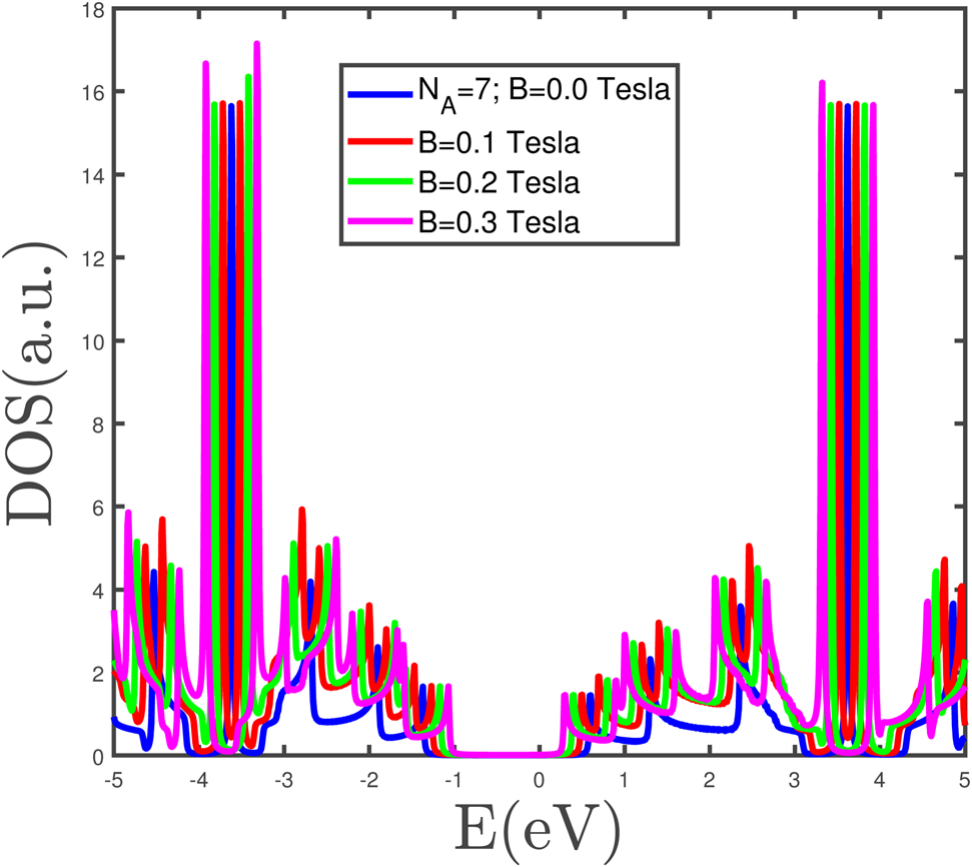
The electronic density of states of the phosphorene armchair nanoribbon with width *w* = 7 for different values of magnetic field. The strain parameter is assumed to be zero.

**Fig. 5:**
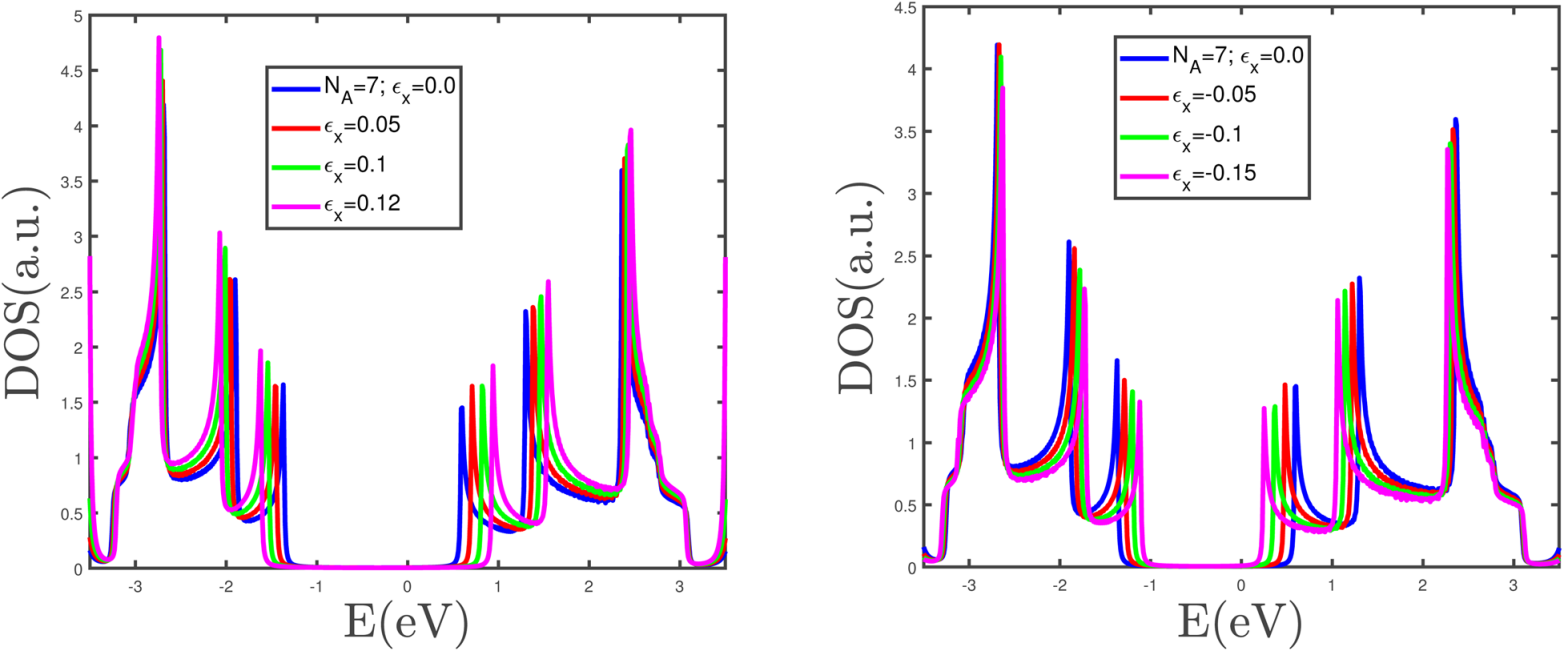
The electronic density of states of the armchair phosphorene nanoribbon with width *w* = 7 for different compressive strains in the right panel and different tensile strains in the left panel. The magnetic field is assumed to be zero.

The band structure of the armchair phosphorene nanoribbon with width *w* = 7 in the absence of a magnetic field and strain parameter has been plotted in Fig. 3. The band gap at wave vector *k*_*x*_ = π/*a* is clearly observed in this figure. This fact confirms the insulating phase for the armchair phosphorene nanoribbon with *w* = 7 at *B* = 0 and *ε* = 0.0. The energy dependence of the density of states of the armchair phosphorene nanoribbon for different values of magnetic field has been plotted in Fig. 4. The strain parameter is fixed at zero. This figure indicates that the band-gap width in the density of states decreases with the magnetic field. However, the variation in the magnetic field preserves the area below the density of states curves, which is because the concentration of electrons does not change with the magnetic field. The behaviors of the density of states of the armchair phosphorene nanoribbon in terms of energy, for different strain parameters and in the absence of an applied magnetic field, have been shown in the panels of Fig. 5. In the right panel, we have plotted the results of the density of states for different compressive strains. This panel shows that the band-gap width decreases with the compressive-strain parameter. However, the left panel of Fig. 5 indicates that an increase in the tensile-strain parameter leads to an enhanced band-gap width in the density of states.

**Fig. 6:**
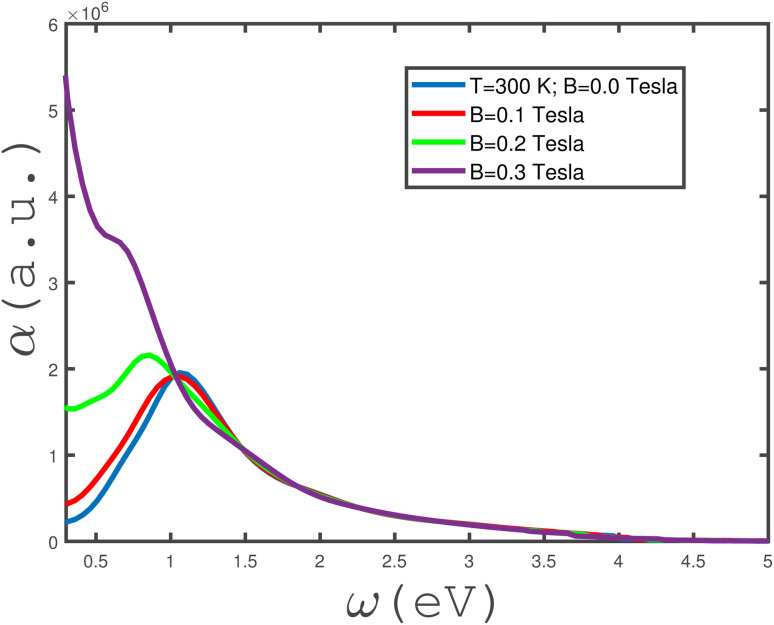
The absorption coefficient, *α*(*ω*), of the undoped armchair phosphorene nanoribbon with width *w* = 7 as a function of incident electromagnetic wave frequency, in the absence of strain values, *i.e.*, *ε*_*x*_ = *ε*_*y*_ = 0, for different values of applied magnetic field at a fixed temperature *T* = 300 K.

The frequency dependence of the absorption coefficient *α*(*ω*) of the undoped armchair phosphorene nanoribbon in the absence of any type of strain for different values of magnetic field has been shown in Fig. 6. Based on this figure, it is clearly observed that the zero-frequency limit of *α*(*ω*) increases with magnetic field. Such a finite value for the zero-frequency limit of *α*(*ω*) comes from an intraband transition of electrons due to their classical behavior in this limit. Also, it is clearly observed that there is a peak at finite non-zero-frequency in *α*(*ω*) for magnetic fields *B* = 0.0, 0.1 and 0.2 Tesla. Mainly, the appearance of these peaks arises from the interband transition of electrons. The frequency peak position tends towards lower values with decreasing magnetic field. This fact can be understood in terms of the band-gap width in the density of states decreasing with magnetic field and thus the curves of the absorption coefficient in Fig. 6 overlap in the electromagnetic wave frequency region *ω* > 1.5 eV.

**Fig. 7:**
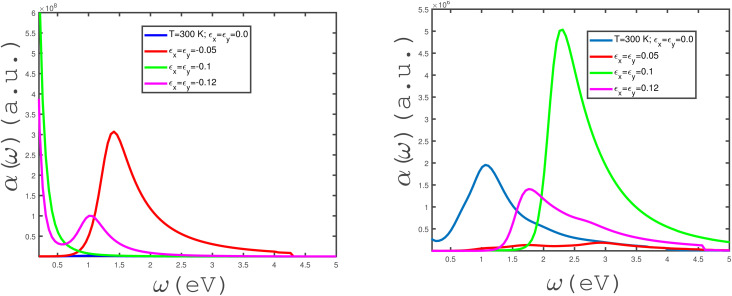
Absorption coefficient, *α*(*ω*), of the undoped armchair phosphorene nanoribbon with width *w* = 7 as a function of photon frequency, in the absence of a magnetic field for different values of in-plane biaxial strain, *ε*_*x*_ ≠ 0, *ε*_*y*_ ≠ 0, at fixed temperature *T* = 300 K. Right panel: with positive strains *ε*_*x*_ = *ε*_*y*_ = 0.0, 0.05, 0.1, 0.12; left panel: with negative strains *ε*_*x*_ = *ε*_*y*_ = 0.0, −0.05, −0.1, −0.12.

We have studied the effects of in-plane biaxial strain, *ε*_*x*_ = *ε*_*y*_, on the frequency dependence of *α*(*ω*) of an undoped armchair phosphorene nanoribbon, as shown in the panels of Fig. 7. The effects of both tensile and compressive biaxial strains have been shown in the panels of this figure. Here, the applied longitudinal magnetic field is assumed to be zero and the temperature has been fixed at *T* = 300 K. In the left panel of Fig. 7, the effects of negative biaxial strain, *i.e.*, compressive strain, on the dependence of *α*(*ω*) on frequency have been shown. According to the left panel of Fig. 7, there is no peak in *α*(*ω*) at compressive-strain parameter *ε*_*x*_ = *ε*_*y*_ = −0.1. The considerable value for the zero-frequency limit of *α*(*ω*) at *ε*_*x*_ = *ε*_*y*_ = −0.1 could be evidence for metallic properties of the structure. Such a high value for the absorption coefficient at the zero-frequency limit at *ε*_*x*_ = *ε*_*y*_ = −0.1 could result from intraband electronic transitions. At compressive-biaxial-strain parameter *ε*_*x*_ = *ε*_*y*_ = −0.05, a peak appears in the absorption coefficient due to the interband transition of electrons in the band structure of the phosphorene nanoribbon. However, *α*(*ω* → 0) vanishes, so this can substantiate the non-metallic behavior of the structure for strain parameter *ε*_*x*_ = *ε*_*y*_ = −0.05. The frequency behavior of *α*(*ω*) at *ε*_*x*_ = *ε*_*y*_ = −0.12 includes a peak with low height at *ω* ≈ 1.0, although the absorption coefficient has a finite value at the zero-frequency limit, as shown in left panel of Fig. 7. The behaviors of the frequency dependence of *α*(*ω*) of the undoped armchair phosphorene nanoribbon for different tensile biaxial strains in the absence of a magnetic field have been presented in the right panel of Fig. 7. The temperature and magnetic field have been assumed to be 300 K and zero, respectively. The absorption coefficient takes a zero value at the zero-frequency limit for all non-zero tensile biaxial strains. Meanwhile, there is a peak in *α*(*ω*) at finite frequency for all values of *ε*_*x*_ = *ε*_*y*_. However, the height of the peak in *α*(*ω*) for *ε*_*x*_ = *ε*_*y*_ = 0.05 is less considerable. Such a peak in the absorption coefficient indicates that interband transition contributes to the electronic transition and consequently the system behaves as a non-metal. According to the right panel in Fig. 7, the intraband transition contributes to electronic transitions in the absence of a strain parameter, which leads to an apparent non-zero value for *α*(*ω* → 0). The armchair phosphorene nanoribbon under all tensile biaxial strains acts as a transparent medium at frequencies *ω* > 5.0 eV, so that the optical absorption has a zero value in this region, as shown in the right panel of Fig. 7. The absorption coefficient of the armchair phosphorene nanoribbon takes the largest value at the peak position for tensile-strain parameter *ε*_*x*_ = *ε*_*y*_ = 0.1.

**Fig. 8:**
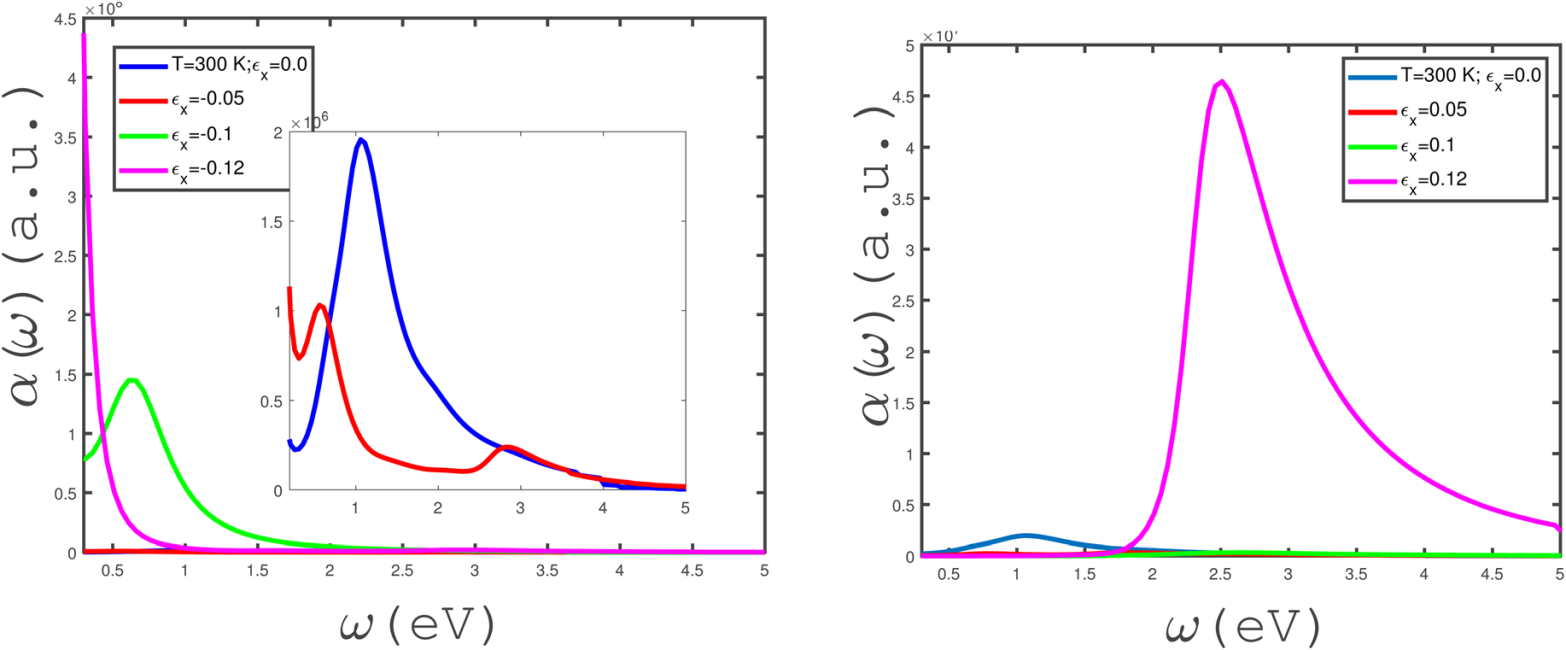
Absorption coefficient, *α*(*ω*), of the undoped armchair phosphorene nanoribbon with width *w* = 7 as a function of photon frequency, in the absence of a magnetic field for different values of in-plane uniaxial strain *ε*_*x*_ ≠ 0 at fixed temperature *T* = 300 K. Left panel: with negative uniaxial strains *ε*_*x*_ = 0.0, −0.05, −0.1, −0.12; right panel: with positive uniaxial strains *ε*_*x*_ = 0.0, 0.05, 0.1, 0.12.

The effects of in-plane uniaxial strain along zigzag direction, *ε*_*x*_, on the behaviors of the absorption coefficient of the undoped armchair phosphorene nanoribbon have been studied, as shown in the panels of Fig. 8. We have plotted the frequency dependence of *α*(*ω*) for different in-plane compressive uniaxial strains, *ε*_*x*_, at zero magnetic field and temperature *T* = 300 eV in the left panel of Fig. 8. The intraband band transition makes a remarkable contribution to the absorption coefficient at the zero-frequency limit for *ε*_*x*_ = −0.12. For this strain parameter, there is no peak at finite frequency, so that a monotonic decreasing behavior is clearly observed for *α*(*ω*) at *ε*_*x*_ = −0.12. Consequently, the interband electronic contribution does not make a contribution to the absorption coefficient at strain *ε*_*x*_ = −0.12. Although the left panel of Fig. 8 indicates that *α*(*ω*) includes a peak at finite non-zero frequency for strain parameters *ε*_*x*_ = 0.0, −0.05, −0.1, the inset shows a peak in the absorption coefficient with very low height in the absence of a strain parameter.

The behavior of *α*(*ω*) in terms of electromagnetic wave frequency for different uniaxial tensile strains, *ε*_*x*_, at fixed temperature *T* = 300 K in the absence of a magnetic field, has been shown in right panel of Fig. 8. Based on this figure, the absorption coefficient takes remarkable values in the frequency region of 2 eV < *ω* < 5 eV at a tensile-strain value *ε*_*x*_ = 0.12. The considerable peak in *α*(*ω*) appears at frequency *ω* = 2.5 eV for *ε*_*x*_ = 0.12. The value of *α*(*ω* → 0) for each uniaxial tensile-strain parameter value, so that the intraband transition does not contribute to the absorption rate of the electromagnetic wave. As shown in the right panel of Fig. 8, the absorption coefficient for *ε*_*x*_ = 0.12 vanishes in the region *ω* < 1.8 eV. Also, it is clearly observed that *α*(*ω*) has no remarkable values in the whole range of frequency at *ε*_*x*_ = 0.1.

**Fig. 9:**
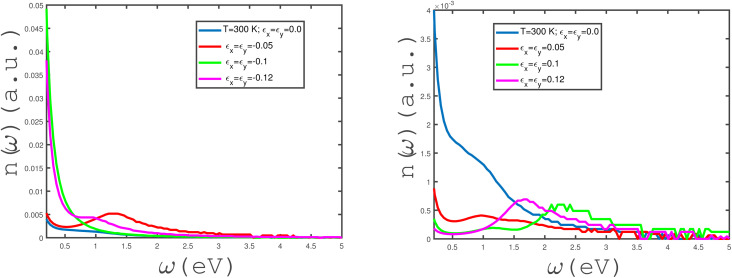
Refractive index, *n*(*ω*), of the undoped armchair phosphorene nanoribbon with width *w* = 7 as a function of photon frequency, in the absence of a magnetic field for different values of in-plane biaxial strain *ε*_*x*_ ≠ 0, *ε*_*y*_ ≠ 0 at fixed temperature *T* = 300 K. Right panel: with positive strains *ε*_*x*_ = *ε*_*y*_ = 0.0, 0.05, 0.1, 0.12; left panel: with negative strains *ε*_*x*_ = *ε*_*y*_ = 0.0, −0.05, −0.1, −0.12.

We have studied the effects of the in-plane biaxial strain, *ε*_*x*_ = *ε*_*y*_, on the frequency dependence of the refractive index, *n*(*ω*), of the undoped armchair phosphorene nanoribbon, as shown in the panels of Fig. 9. Both tensile and compressive biaxial strain effects have been shown in the panels of this figure. Here, the applied longitudinal magnetic field is assumed to be zero and the temperature has been fixed at *T* = 300 K. In the left panel of Fig. 9, the effects of compressive biaxial strain on the dependence of the refractive coefficient on frequency have been shown. According to the left panel of Fig. 9, the zero-frequency limit of *n*(*ω*) has considerable values at compressive-strain parameters *ε*_*x*_ = *ε*_*y*_ = −0.1, −0.12. At compressive-biaxial-strain parameter *ε*_*x*_ = *ε*_*y*_ = −0.05, a peak appears in the refractive index of the phosphorene nanoribbon at frequency *ω* = 1.25 eV. The zero-frequency limit of *n* has remarkable values for *ε*_*x*_ = *ε*_*y*_ = −0.1, −0.12 in comparison with the other strain parameters, according to left panel of Fig. 9. The dependence of *n*(*ω*) on the frequency shows a monotonic decreasing behavior for biaxial compressive-strain parameters *ε*_*x*_ = *ε*_*y*_ = 0.0, −0.1, −0.12. Moreover, the refractive index *n*(*ω*) goes to zero for all compressive strains in the frequency region *ω* > 2.5 eV. The behaviors of the frequency dependence of the refractive index *n*(*ω*) of the undoped armchair phosphorene nanoribbon for different tensile biaxial strains, in the absence of a magnetic field, have been presented in the right panel of Fig. 9. The temperature has been assumed to be *T* = 300 K. The zero-frequency limit of *n*(*ω*) decreases with the increase in the absolute value of the tensile-strain parameter. Meanwhile, there is a peak in *n*(*ω*) at finite frequency for tensile-strain values *ε*_*x*_ = *ε*_*y*_ = 0.05, 0.1, 0.12. The frequency dependence of the refractive index of the armchair phosphorene nanoribbon shows a monotonic decreasing behavior in the absence of a tensile-biaxial-strain parameter, as shown in the right panel of Fig. 9.

**Fig. 10:**
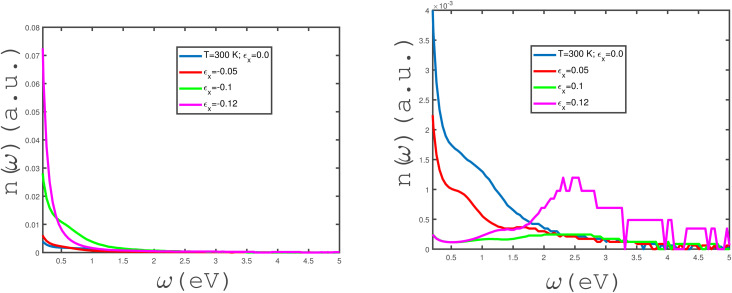
Refractive index, *n*(*ω*), of the undoped armchair phosphorene nanoribbon with width *w* = 7 as a function of photon frequency, in the absence of a magnetic field for different values of in-plane uniaxial strain *ε*_*x*_ ≠ 0 at fixed temperature *T* = 300 K. Left panel: with negative uniaxial strains *ε*_*x*_ = 0.0, −0.05, −0.1, −0.12; right panel: with positive uniaxial strains *ε*_*x*_ = 0.0, 0.05, 0.1, 0.12.

The effects of in-plane uniaxial strain along the zigzag direction, *i.e.*, *ε*_*x*_, on the behaviors of the refractive index of the undoped armchair phosphorene nanoribbon have been studied, as shown in the panels of Fig. 10. We have plotted the frequency dependence of the refractive index, *n*(*ω*), for different values of in-plane compressive uniaxial strain, *ε*_*x*_, in the left panel of Fig. 10. The temperature has been fixed at *T* = 300 K and the applied magnetic field is assumed to be zero. For each compressive uniaxial strain, *ε*_*x*_, it is clearly observed that the refractive index decreases monotonically in terms of electromagnetic wave frequency. Meanwhile, the zero-frequency limit of *n*(*ω*) increases with an enhancement in the absolute values of *ε*_*x*_ according to the left panel of Fig. 10. The behavior of *n*(*ω*) in terms of frequency, *ω*, for different uniaxial tensile strains, *ε*_*x*_, at fixed temperature has been shown in the right panel of Fig. 10. The temperature and magnetic field strength have been fixed at *T* = 300 K and *B* = 0 Tesla, respectively. The zero-frequency limit of the refractive index decreases with the tensile-strain parameter, *ε*_*x*_; however, *n*(*ω*) at the zero-frequency limit has the same values for strain parameters *ε*_*x*_ = 0.1, 0.12. The refractive index, *n*, includes a peak at finite frequency *ω* ≈ 2.5 eV for *ε*_*x*_ = 0.12, in contrast to the other tensile-uniaxial-strain parameters.

**Fig. 11:**
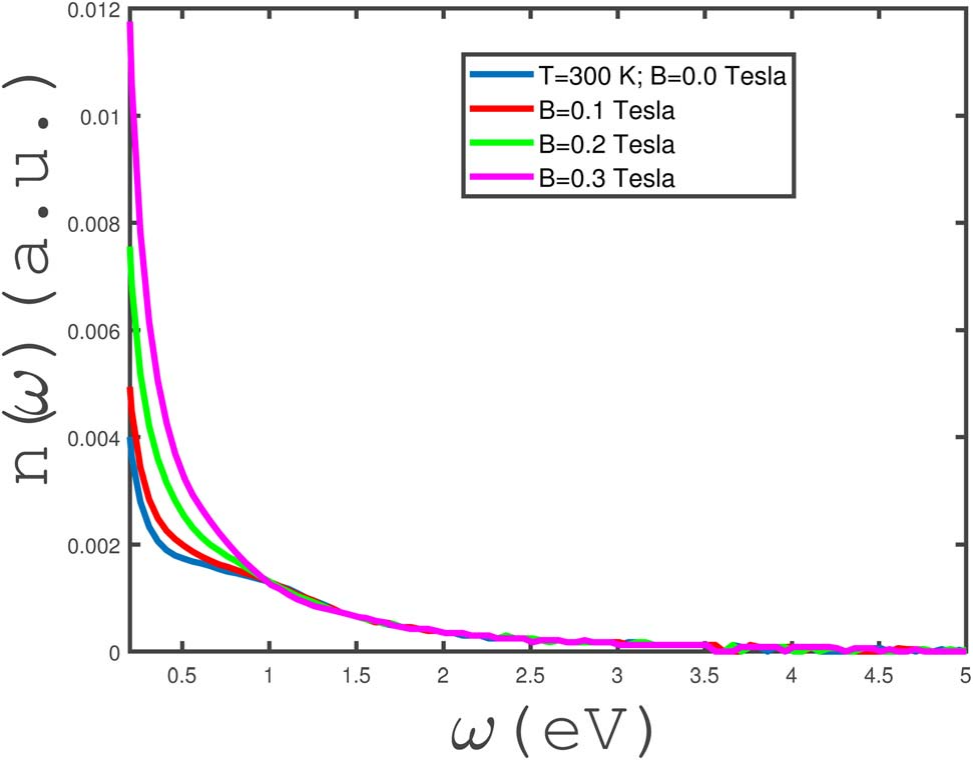
Refractive index, *n*(*ω*), of the undoped armchair phosphorene nanoribbon with width *w* = 7 as a function of photon frequency in the absence of strain values, *i.e. ε*_*x*_ = *ε*_*y*_ = 0, for different values of applied magnetic field at fixed temperature *T* = 300 K.

The frequency dependence of the refractive index, *n*(*ω*), of the undoped armchair phosphorene nanoribbon in the absence of any type of strain for different values of magnetic field has been shown in Fig. 11. *n*(*ω* → 0) of the undoped unstrained armchair phosphorene nanoribbon takes non-zero values for all magnetic fields. Based on this figure, it is clearly observed that the zero-frequency limit of the refractive index increases with magnetic field. In addition, at fixed frequency below 1.0 eV, the refractive index, *n*(*ω*), increases with magnetic field, *B*; however, the curves of the refractive index overlap for all magnetic fields in the frequency region *ω* > 1.0 eV. A monotonic decreasing behavior for the frequency dependence of *n*(*ω*) is clearly observed for each magnetic field based on Fig. 11.

**Fig. 12:**
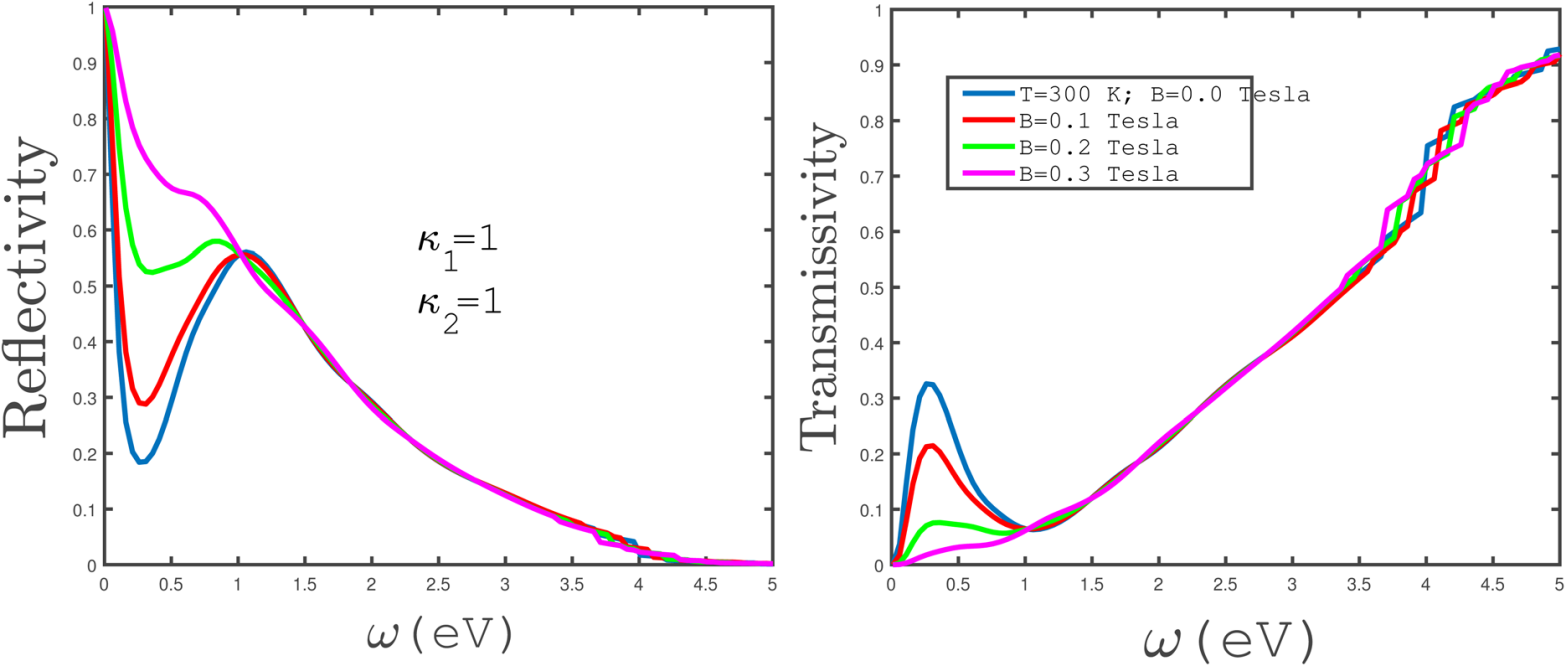
The frequency dependence of the reflectivity (left panel) and transmissivity (right panel) of electromagnetic waves between two media with permittivities *κ*_1_ = *κ*_2_ = 1 separated by an undoped armchair phosphorene nanoribbon with width *w* = 7. The magnetic field is varied as *B* = 0.0, 0.1, 0.2, 0.3 Tesla. The strain parameter is assumed to be zero and the temperature has been fixed at *T* = 300 K.

In the panels of Fig. 12, we have plotted the frequency behavior of the transmissivity and reflectivity of electromagnetic waves between two media separated by an unstrained undoped armchair phosphorene nanoribbon for normal incidence. Both media are vacuums with electrical permittivities *κ*_1_ = *κ*_2_ = 1. The left panel of Fig. 12 presents the frequency dependence of the reflection coefficient of the incident electromagnetic wave for different values of magnetic field at fixed temperature *T* = 300 K. The zero-frequency limit of the reflection coefficient has the same value, one, for all values of magnetic field. For applied magnetic field *B* = 0.3 Tesla, the frequency dependence of the reflectivity shows a monotonically decreasing behavior. With a reduction in the magnetic field, a valley and a peak appear in the reflection coefficient at magnetic fields *B* = 0.0, 0.1, 0.2 Tesla according to the left panel of Fig. 12. The frequency positions of the valleys in the reflection coefficient are *ω* ≈ 0.35 eV. The positions of the valleys are independent of magnetic field; however, the depth of the valleys decreases with magnetic field. The position of the peak in the reflection coefficient is around 1.2 eV for all magnetic fields and the height is not affected by the strength of *B*. Moreover, the curves of the frequency dependence of reflectivity overlap in the region *ω* > 1.5 eV for all magnetic field strengths. The electromagnetic wave frequency dependence of the transmission coefficient of the undoped unstrained armchair phosphorene nanoribbon, for different values of magnetic field, has been plotted in the right panel of Fig. 12. For applied magnetic field *B* = 0.3 eV, an increasing behavior for the frequency dependence of the transmissivity is clearly observed. A peak appears in the transmission coefficient of the phosphorene nanoribbon at *ω* ≈ 0, 25 eV for magnetic fields *B* = 0.0, 0.1, 0.2 Tesla, although the height of the peak decreases with magnetic field according to the right panel of Fig. 12. Also, we find an increasing behavior for the frequency dependence of transmission in the region *ω* > 1.0 eV for all values of magnetic fields. Moreover the results of the transmissivity curves overlap for different magnetic fields in this frequency region.

**Fig. 13:**
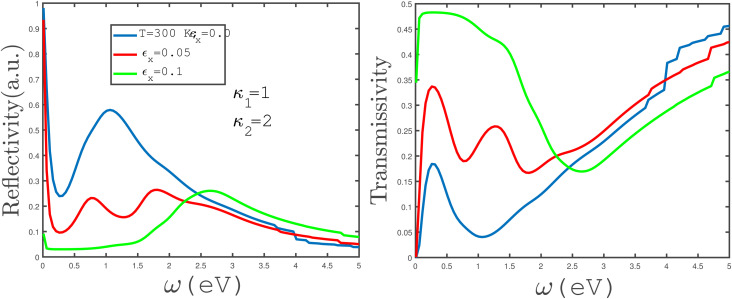
The frequency dependence of the reflectivity (left panel) and transmissivity (right panel) of electromagnetic waves between two media with permittivities *κ*_1_ = 1 and *κ*_2_ = 2, separated by an undoped armchair phosphorene nanoribbon with width *w* = 7. The magnetic field has a zero value. The tensile-strain parameter is varied in both panels and the temperature has been fixed at *T* = 300 K.

In Fig. 13, the left panel shows the frequency dependence of the reflection coefficient of electromagnetic waves between two media separated by an armchair phosphorene nanoribbon. Uniaxial tensile strain with amounts *ε*_*x*_ = 0.0, 0.05, 0.1 has been applied to the phosphorene ribbon and the temperature has been fixed at 300 K. As we can see in this figure, increasing the tensile strain in the frequency range of 0.0 eV < *ω* < 2.25 eV causes a decrease in the reflection coefficient, while at a tensile strain of *ε*_*x*_ = 0.05, it leads to two peaks appearing in the reflection coefficient of the phosphorene nanoribbon armchair. The transmissivity coefficient under uniaxial tensile strain at a constant temperature of 300 K is plotted for two different media with electrical permittivities *κ*_1_ = 1 and *κ*_2_ = 2 for different tensile uniaxial-strain values, *ε*_*x*_ = 0.0, 0.05, 0.1, in the right panel of Fig. 13. As can be observed in the figure, in the frequency range of 0.0 eV < *ω* < 2.25 eV, an increase in uniaxial tensile strain gives rise to an increase in the transmissivity coefficient. Also, for uniaxial tensile strain *ε*_*x*_ = 0.05, two peaks have appeared in the transmissivity coefficient curve.

**Fig. 14:**
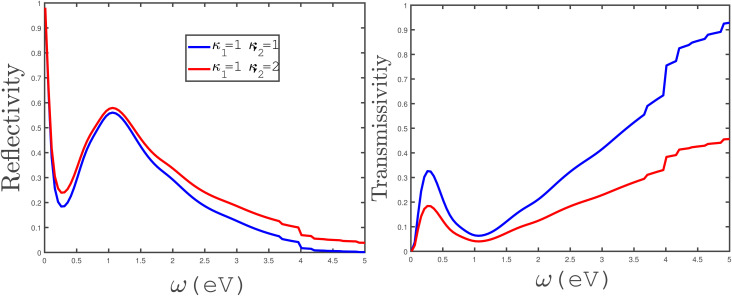
The frequency dependence of the reflectivity (left panel) and transmissivity (right panel) of electromagnetic waves between two media with permittivities *κ*_1_ = 1 and *κ*_2_ = 1 or 2, separated by the undoped armchair phosphorene nanoribbon with width *w* = 7. The magnetic field has a zero value. The tensile-strain parameter value is varied in both panels and the temperature has been fixed at *T* = 300 K.

In the panels of Fig. 14, the effects of variation of the electrical permittivity of the second medium on the reflectivity and transmissivity of electromagnetic waves by the armchair phosphorene nanoribbon have been shown. We have plotted the reflection coefficient in terms of incident electromagnetic wave frequency in the absence of an applied magnetic field for different values of the permittivity of the second medium, *κ*_2_ = 1, 2. The permittivity of the first medium has been fixed at *κ*_1_ = 1. According to the left panel of Fig. 14, the reflectivity of electromagnetic waves by the phosphorene nanoribbon increases with the permittivity of the second medium. However, the reflection coefficient is approximately independent of the second medium at frequencies *ω* < 0.25 eV. Also, a peak in the reflectivity appears at *ω* = 1.0 eV for both electrical permittivities *κ*_2_ = 1, 2. In the right panel of Fig. 14, the transmissivity of electromagnetic waves by the phosphorene nanoribbon has been plotted in terms of frequency for the two different permittivities of the second medium. The results in this panel show that the transmissivity of electromagnetic waves by the phosphorene nanoribbon decreases with the permittivity of the second medium.

Here we mention some points about the main advantages of this armchair phosphorene nanoribbon material and the novelty of this work. Jain *et al.*, by using DFT and density functional perturbation theory (DFPT) computations, predicted that phosphorene monolayers would have thermal conductivities of 36 and 110 W mK^−1^ along the *x* and *y* directions, respectively, at 300 K. The comparable thermal conductivity to that of MoS_2_ makes phosphorene attractive for thermal transport.^82^ Moreover, Fei *et al.* predicted that phosphorene also possesses an anisotropic electrical conductance that is oriented orthogonally to the anisotropic thermal conductance, thus resulting in a high ratio of electrical conductance to thermal conductance for phosphorene.^83^ As a consequence, phosphorene can convert heat energy to electrical energy with high efficiency, which is desirable for thermoelectrics. Also, some of the theoretically predicted electronic and optical properties of phosphorene, besides its carrier mobility, have been well verified by experimental investigations. For example, Zhang *et al.* revealed that few-layer phosphorene shows strong and layer-dependent photoluminescence (PL) and an anisotropic Raman response.^84^ They demonstrated that the PL peaks show a blue shift with increasing layer thickness, indicating consistent results with those of computations, and experimentally verifying the thickness-dependent band gap of phosphorene. Moreover, by performing linearly polarized Raman measurements, it was demonstrated that the anisotropic Raman response can be utilized to quickly determine the crystalline orientation of phosphorene, echoing the theoretical prediction.^85^ The electronic transport and optical properties of phosphorene nanoribbons with different edges, such as zigzag or armchair, have extensive applications in future nano-electronics,^86^ and phosphorene is of great interest in engineering applications because of its extraordinary electronic and optical properties. So far, no study has been carried out on the properties of the optical conductivity, imaginary and real parts of the dielectric constant, and optical coefficients of armchair phosphorene nanoribbons under uniaxial and biaxial strains. In this manuscript, we investigated the optical properties of armchair phosphorene nanoribbons using the Kane–Mele model. This study is expected to play an important role in the creation of phosphorene devices.

Some statements regarding the importance of the dielectric and magnetic properties of phosphorene layers for EM absorption are suitable here. In this work, we have applied a magnetic field perpendicular to the plane of the phosphorene nanoribbon. Such an applied magnetic field leads to magnetic ordering and the effects of that on the optical properties of phosphorene are studied. However, the effects of spontaneous magnetic long-range ordering on the optical properties of the sample are not the main aim of this study. Meanwhile, we deal with a very thick phosphorene layer, so that the study of skin depth cannot be discussed here. However, we can readily find the skin depth, *i.e.*, *δ*(*ω*), for the bulk system according to the relation 
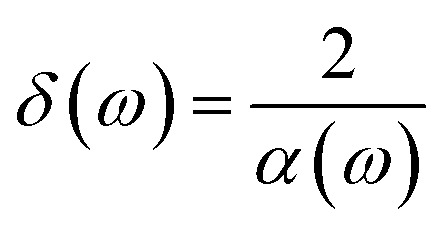
, where the absorption coefficient, *α*(*ω*), is calculated according to eqn (22). In the case of impedance, we can only mention at this point that such a parameter is found based on the inversion of dynamic optical conductivity, *σ*(*ω*), which has been given by eqn (18).

## Author contributions

H. Rezania, M. Abdi, E. Nourian and B. Astinchap contributed equally to the paper in the view point of writing and mathematical calculations.

## Conflicts of interest

There is no conflicts to declare.

## Supplementary Material
